# What Online Communities Can Tell Us About Electronic Cigarettes and Hookah Use: A Study Using Text Mining and Visualization Techniques

**DOI:** 10.2196/jmir.4517

**Published:** 2015-09-29

**Authors:** Annie T Chen, Shu-Hong Zhu, Mike Conway

**Affiliations:** ^1^ School of Information and Library Science University of North Carolina at Chapel Hill Chapel Hill, NC United States; ^2^ Department of Family Medicine and Public Health University of California, San Diego La Jolla, CA United States

**Keywords:** electronic cigarettes, hookah smoking, cigarettes, tobacco products, social media, text mining

## Abstract

**Background:**

The rise in popularity of electronic cigarettes (e-cigarettes) and hookah over recent years has been accompanied by some confusion and uncertainty regarding the development of an appropriate regulatory response towards these emerging products. Mining online discussion content can lead to insights into people’s experiences, which can in turn further our knowledge of how to address potential health implications. In this work, we take a novel approach to understanding the use and appeal of these emerging products by applying text mining techniques to compare consumer experiences across discussion forums.

**Objective:**

This study examined content from the websites Vapor Talk, Hookah Forum, and Reddit to understand people’s experiences with different tobacco products. Our investigation involves three parts. First, we identified contextual factors that inform our understanding of tobacco use behaviors, such as setting, time, social relationships, and sensory experience, and compared the forums to identify the ones where content on these factors is most common. Second, we compared how the tobacco use experience differs with combustible cigarettes and e-cigarettes. Third, we investigated differences between e-cigarette and hookah use.

**Methods:**

In the first part of our study, we employed a lexicon-based extraction approach to estimate prevalence of contextual factors, and then we generated a heat map based on these estimates to compare the forums. In the second and third parts of the study, we employed a text mining technique called topic modeling to identify important topics and then developed a visualization, Topic Bars, to compare topic coverage across forums.

**Results:**

In the first part of the study, we identified two forums, Vapor Talk Health & Safety and the Stopsmoking subreddit, where discussion concerning contextual factors was particularly common. The second part showed that the discussion in Vapor Talk Health & Safety focused on symptoms and comparisons of combustible cigarettes and e-cigarettes, and the Stopsmoking subreddit focused on psychological aspects of quitting. Last, we examined the discussion content on Vapor Talk and Hookah Forum. Prominent topics included equipment, technique, experiential elements of use, and the buying and selling of equipment.

**Conclusions:**

This study has three main contributions. Discussion forums differ in the extent to which their content may help us understand behaviors with potential health implications. Identifying dimensions of interest and using a heat map visualization to compare across forums can be helpful for identifying forums with the greatest density of health information. Additionally, our work has shown that the quitting experience can potentially be very different depending on whether or not e-cigarettes are used. Finally, e-cigarette and hookah forums are similar in that members represent a “hobbyist culture” that actively engages in information exchange. These differences have important implications for both tobacco regulation and smoking cessation intervention design.

##  Introduction

In recent years, researchers have begun to realize the value of social media (including online discussion forums) as a data source for understanding health-related phenomena. The pervasiveness, ubiquity, and real-time nature of social media makes it useful for biosurveillance applications such as mining for influenza mentions, as well as studies of information dissemination and public sentiment towards topics such as vaccination [1-3]. Various terms have been used to describe this new and growing field, including infodemiology [4], digital disease detection [5], and digital epidemiology [6]. Moreover, social media mining has also been employed to understand the public’s impression of products that have health implications [7]. The content of health discussion forums can provide rich details concerning the context in which patients experience various health issues, including temporal and emotional factors, which may help us tailor information to fit their needs [8]. In recent years, there has been increased interest in leveraging the use of online social networks for interventions to promote population-level smoking cessation [9].

This study is focused on leveraging the rich detail that is often provided in discussion forums to understand more about the experiences of users of three tobacco products—combustible cigarettes, electronic cigarettes (e-cigarettes), and water pipes (also known as “hookah”)—and their potential health implications. E-cigarettes have increasingly gained popularity, particularly in those markets with well-developed tobacco control policies like the United States and (parts of) the European Union [10-12]. Current smokers and tobacco users are more likely to try e-cigarettes than those who have never smoked or used tobacco [12]. Dual use of e-cigarettes and combustible cigarettes is common among smokers who are considering quitting in the next 6 months [13].

Previous literature has enumerated various motivations for e-cigarette use, including quitting smoking for health reasons, the belief that e-cigarettes are safer than regular cigarettes, e-cigarettes are cheaper than regular cigarettes, e-cigarettes are allowed in locations where regular cigarettes are not, avoiding disturbing others with secondhand smoke, the sheer pleasure of smoking, and “just because” [12,14]. Reasons for stopping use included users thinking they did not need them anymore or that they would not relapse to smoking if they stopped, poor product quality, no reduction in cravings, relapse to smoking, and the lack of efficacy in helping users to quit smoking [15].

Aside from e-cigarettes, there has been increasing concern about the growing use of hookah (also known by other names such as waterpipe, shisha, and hubble bubble) worldwide [16]. Hookah is a centuries old practice that experienced a resurgence in the Middle East in the 1990s [17]. A hookah consists of a bowl where the burning tobacco is placed, an ashtray, stem, air valve, water base, and one or more hoses and mouthpieces. During use, smoke from the burning tobacco descends to the bowl of water that it bubbles through and is then inhaled by the smoker through a mouthpiece.

Hookah use is often a social behavior, and hookah bars or lounges appear to play an important role in the increased popularity of hookah smoking [18]. Aspects of group use such as group size and the number of waterpipes available per table may affect toxicant exposure; thus, it is important to consider the social and contextual factors associated with use [19]. Factors that have contributed to the rise in hookah use include availability in cafes and restaurants, social aspects, affordability, appeal of hookah designs, sensory aspects of the hookah smoking experience, and media influence [20]. Predictors of hookah use include current and past cigarette use, and alcohol and marijuana use [21-23].

Use of hookah may have various negative health effects, for example, developing chronic obstructive pulmonary disease and chronic bronchitis, increased risk of lung cancer and esophageal cancer, and adverse effects on cardiovascular health [24]. However, previous research suggests that hookah users believe that hookah is less harmful than traditional cigarettes, and thus the argument has been made that there is a greater need for education concerning the potential health dangers of hookah use [22,25].

Though there is a considerable research currently being undertaken on the health effects of e-cigarettes and hookah, there is less work focused on how people are using these tobacco products in naturalistic settings. However, in recent years, there have been a number of studies that have investigated e-cigarette and hookah mentions in social media, including symptoms that were reported by participants in three discussion forums [26], sentiment towards e-cigarette and hookah use on Twitter [4], marketing of electronic cigarettes on Twitter [27], hookah references on Facebook profiles of American college students [28], and e-cigarette and hookah videos on YouTube [29].

There are many different kinds of social media, and it can be problematic to employ social media data from a single source, or even multiple sources, to make population-level inferences [30]. This is not what we endeavor to do in this study. Rather, we demonstrate methods that can be used to compare across data sources and mitigate the effects of source differences, to make inferences about the sample that is being studied. We also try to provide enough contextual detail to enable readers to understand the extent to which the results may be applicable to other populations and to generate hypotheses for future research. In this study, we used several data sources to understand how different online communities might address the same topic.

As far as we know, there has not been a text mining study that has taken a comparative approach to examining online communities and tobacco products, and more specifically, examining what the discussion content may suggest about the appeal and motivation for use. With this study, we have endeavored to fill that gap. We selected multiple online communities, in order to develop a better sense of the diversity of online content with these products. We focused on six different discussion forums on three different websites: Vapor Talk, Hookah Forum, and Reddit. We expected that these samples might differ on a variety of characteristics and thus serve as an appropriate set of samples for comparison.

This study is structured into three distinct parts. In the first part, we employed a heat map visualization to compare different aspects of e-cigarette and hookah use behavior across multiple forums to identify the forums with the highest concentration of reports concerning social and contextual factors of e-cigarette and hookah use, including the settings where use behaviors occur (eg, restaurant, lounge, and party), time, social relationships, and sensory experience. The heat map facilitated a quick visual scan enabling us to determine which discussion forums might contain the richest discussions of behavior relevant to e-cigarette and hookah use, and thus, enabled selection of data subsets for further analyses.

In the second part, we integrated text mining and visualization techniques to render a visualization, Topic Bars, to compare discussion content in two forums: the Health & Safety forum on Vapor Talk, which is focused on e-cigarettes, and the Stopsmoking subreddit, which is primarily concerned with quitting traditional, combustible cigarettes (analogs).

In the third and last part, we compare experiences with e-cigarette and hookah use. How does the nature of content on these two products differ? We examined this question through a Topic Bars visualization depicting the general discussion forums for Vapor Talk (focused on e-cigarettes) and Hookah Forum (focused on hookah).

## Methods

### Harvesting of the Document Collection

We downloaded publicly available content from three websites: (1) Vapor Talk [31], a forum dedicated to e-cigarettes, (2) Hookah Forum [32], and (3) Reddit [33], a platform that hosts subforums or “subreddits” on a wide variety of topics.

Vapor Talk and Hookah Forum are online communities that are dedicated to e-cigarettes and hookah, respectively. At the time the data were collected, Vapor Talk and Hookah Forum appeared among the top results on the Google search engine when searching using keywords such as “e-cigarette”, “vaping”, “hookah”, “health”, and “forum”. Vapor Talk has also been examined in previous research [26]. Vapor Talk features a number of different forums; we selected “General E-Cig Discussion” and “Health & Safety.” These two forums were selected to acquire a general sense of what the nature of discussion concerning e-cigarettes is like, as well as the community’s specific health concerns.

Reddit is a generic platform featuring “subreddits” on a broad range of topics. The platform is more popular among younger people [34,35]. On Reddit, we examined the “stopsmoking”, “electronic_cigarette”, and “hookah” subreddits.

Publicly available content for each discussion forum was downloaded using a Web crawler, Wget, between April and June 2014. Crawls of each site focused on the discussion content, and no explicit attempt was made to crawl user profiles. The pages of discussion content from Vapor Talk and Hookah Forum include some basic user metadata such as username, gender, and member level. The post content and metadata were extracted using Python code and inserted into a MySQL database.

### Comparing Contextual Factors of Tobacco Use Across Datasets

In this study, we were interested in using social media to understand more about differences in people’s experiences and motivations for using e-cigarettes and hookah, as an understanding of how consumers use different tobacco products is vital for both advancing tobacco regulatory science and smoking cessation intervention design. We identified a set of factors to use to compare across datasets. Understanding the factors that influence people’s behavior can be invaluable for developing strategies to encourage more healthful behaviors. Previous literature has argued that an individual’s behavior is affected by a variety of individual and social factors, including an individual’s beliefs, social interactions, and organizational and policy factors [36]. In addition, factors such as space and time are often critical aspects of health context [37].

These factors include health perceptions about the safety of e-cigarettes versus smoking, cost, sensory pleasure, effect on social relations (eg, not inconveniencing others), and popularity in social settings. We classified these by three main categories of interest: (1) subject matter (e-cigarette and hookah), (2) health (symptoms, quitting, health perceptions, and health care practitioners), and (3) context (social relationships, setting, time, cost and sensory experience). We employed lexicons containing words that represented these categories. By using these words to match against the online discussion content, we could come to understand to what degree the discussion content contained information about these categories of interest. The higher the proportion of this content, the more we might be interested in examining the content in that forum. Table 1 depicts the categories, their definitions, and example terms. The terms in the lexicon are provided in Multimedia Appendix 1.

The process of lexicon development was a hybrid one consisting of both a literature review and iterative testing involving examination of the discussion content. The Symptoms and Quitting terms primarily came from the empirical literature but were augmented using online consumer-generated content, such as guides written for novice users, discussion forums, and websites advertising e-cigarette and hookah products. The other dimensions were primarily drawn from user-generated content and supplemented using empirical research. Lexicon development was an iterative process of adding keywords until the addition of new keywords did not result in substantive differentiation across the datasets being compared.

**Table 1:** Contextual factors of tobacco use: Lexicon definitions and examples.

Contextual factors		Definition
**Subject matter**		
	E-cigarette	The types and parts of e-cigarettes, eg, ecig, vape, “atty” (atomizer), “carto” (cartomizer).
	Hookah	The types and parts of hookahs, eg, hookah, waterpipe, shisha, mouthpiece.
**Health**		
	Symptoms	This set of concepts was constructed from existing literature on the health effects of e-cigarette and hookah use, particularly [26], and also through examination of the discussion content harvested in this study, eg, throat, cough, migraine, craving.
	Quitting	Pertaining to experience of quitting, including motivations (eg, “stigma” and “stink”), difficulties in quitting (eg, “stress”), and tobacco cessation aids; also includes psychological factors such as “depression” and “anxiety”.
	Perceptions	Perceptions of the safety of and potential health implications of e-cigarettes and hookah use, eg, toxic, dangerous, safe.
	Health care practitioners	Various types of health care practitioners, eg, doctors, physicians, therapists, counselors.
**Context**		
	Social relationships	Social relations that are often mentioned in discussion forums, eg, family, friends, children.
	Setting	Settings where vaping and hookah use may occur, eg, home, bar, party.
	Time	Timing of e-cigarette and hookah use, eg, morning, afternoon, evening.
	Cost	Cost aspects of tobacco use, eg, cheap, expensive, price, saving.
	Sensory experience	Sensory aspects of tobacco use, eg, hit, cloud, buzz.

We used these lexicons to estimate the prevalence of each category of interest, and then we rendered a heat map visualization to compare across forums. Heat maps are often used in genetics to display gene expression patterns [38,39] or to show the results of hierarchical clustering. In a classic cluster heat map, one axis of the heat map might represent samples, and the other, genes [40]. Each cell is colored based on the level of expression of the gene in the corresponding sample.

### Topic Modeling and Visualization

In the second and third parts of our study, we used topic models to compare the content of online discussion forums. To model topics, we used a generative probabilistic modeling algorithm, Latent Dirichlet Allocation (LDA). LDA is a technique that models documents as random mixtures over topics, where a topic is characterized as a distribution of words [41].

We employed the LDA implementation that is available with the MALLET toolkit [42]. Previous research has observed that results with and without stemming yield comparable results and that stemmed results are more difficult to interpret [43]. In this study, we opted not to stem because viewing the original versions of the words facilitated interpretation of the context in which words were used. We used an augmented stop word list that included the original MALLET stop word list, as well as other common online forms of non-substantive words and word fragments, such as “ill” (“I’ll) and “dont” and forum members’ usernames. The augmented stop words, with the exception of forum members’ usernames, have been provided in Multimedia Appendix 2.

We trained topic models for four forums: Vapor Talk General E-Cig Discussion, Hookah Forum General Discussion, Vapor Talk Health & Safety, and the Stopsmoking subreddit. We experimented with different numbers of topics in order to find a level of granularity that showed the diversity of discussion topics, while at the same time avoiding topics that were thematically similar. We named all of the topics through a combination of examining keywords and manual examination of posts that were representative of those topics. To reduce complexity, we then grouped these topics together into categories if they were thematically similar. A list of all the topics and their respective categories, for each topic model, is available in Multimedia Appendix 3.

The output of topic modeling includes a set of topics and the main words associated with that topic, as well as a list of documents, with estimates of the proportion of each topic present in each document. Thus, from these outputs, one could say, for example, that if 60% of document A consists of topic X, then document A primarily consists of topic X, with trace amounts of all other topics. Similarly, a document B that is predicted to be 30% topic Y and 30% topic Z might be said to primarily consist of topics Y and Z, with trace amounts of all other topics. One final example would be that a document contains small amounts of all the topics but is not that representative of any topic in particular.

In order to summarize the prevalence of the topics generated, we used an estimate of main “document-topics”. By document topic, we refer to the instances where a topic is a major constituent of a given document. A topic was considered a major constituent of a document if it was predicted to constitute a given minimum proportion of that document. The thresholds were determined by iteratively testing different candidate values until the number of “document-topics” was close to the number of total posts in the discussion forum. The selection of this criterion was to maximize the proportion of content that was represented.

We calculated the number of document-topic elements for each topic and then divided by the number of total document-topic elements, to determine the proportion of a forum that was constituted by each topic. We then used these proportions to render a horizontal stacked bar chart, which supports a visual comparison of topic prevalence within and across discussion forums.

### Research Ethics Statement

Publicly available social media content can be an invaluable complement to data provided by study participants in more explicit research contexts because it is a rich source of information on how behaviors with health impacts may naturally occur in the real world. In order to protect the identities of forum users, we have not provided explicit quotations, but instead described the content in as much detail as possible, both quantitatively and qualitatively, in line with ethical guidelines [44,45]. The work reported in this paper has been certified as exempt from review under 45 CFR 46.101(b), category 4 by the University of California San Diego Institutional Review board (Project #140844X).

## Results

### Harvesting of the Document Collection

We examined content from three different websites: (1) Vapor Talk, a website devoted to e-cigarettes, (2) Hookah Forum, a forum devoted to hookah use, and (3) Reddit, a site featuring discussion forums on a wide variety of topics. On Reddit, we chose to focus on three different discussion forums: “electronic_cigarette”, “hookah”, and “stopsmoking”. On Vapor Talk, we focused on two subforums: “General E-cig Discussion” and “Health & Safety.” On Hookah Forum, we focused on the general discussion forum only, as this website does not have a forum dedicated specifically to health topics. The forums differed considerably in terms of the number of total posts, the mean number of users, and mean post length (Table 2).

**Table 2:** Corpus statistics.

	Vapor Talk		Hookah Forum	Reddit		
	General	Health	General	Stop-smoking	Electronic cigarette	Hookah
Posts, n	11,438	2376	17,761	2092	89,119	43,501
Threads, n	690	172	413	177	2093	2994
Users, n	773	423	1659	760	14,277	4374
Post length, mean (SD)	356.35 (447.33)	487.39 (653.45)	323.16 (520.97)	267.49 (441.77)	189.29 (378.29)	155.88 (263.75)

### Comparing Contextual Factors of Tobacco Use Across Datasets

**Figure 1:**
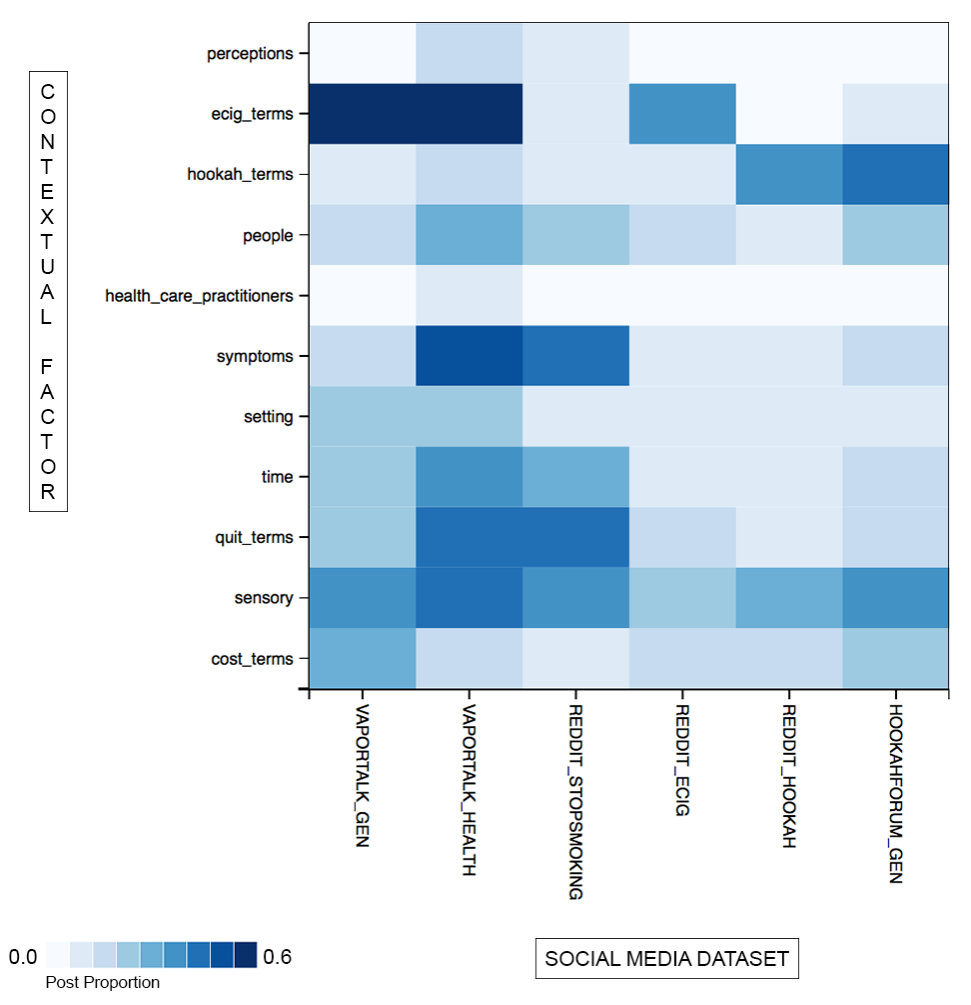
Contextual factors of e-cigarette and hookah use.

In our first research question, we asked what differences there were in the prevalence of contextual factors of e-cigarette and hookah use across different online communities. The prevalence of contextual factors was calculated as the proportion of posts containing a term from the relevant contextual factor lexicon, and a heat map was rendered based on these prevalence estimates (Figure 1). The darker the hue, the higher the proportion of that type of content in the forums, with the darkest cells representing approximately 60% of the forum content.

As we might expect, e-cigarette–related content was most popular in the Vapor Talk forums and on the Electronic_cigarette subreddit, and hookah content was most popular in the hookah forums. The two general forums on Vapor Talk and Hookah Forum contained more content on the cost and purchasing of equipment. Examination of the content showed an active discussion of the “ins and outs” of these products (ie, the detailed description of the intricacies of product use) and cost implications of product use. Descriptions of sensory experience appear common in most of the forums, which suggests that the sensory aspects of use are important across multiple types of tobacco products.

The purpose of the heat map visualization was to identify forums that contained the richest information about contextual factors in e-cigarette and hookah use. We saw that the mentions of people, symptoms, time, quitting, and sensory experience were highest in density in the Vapor Talk Health & Safety forum and in the Stopsmoking subreddit. Examining the discussion content, we saw that a substantial part of this discussion addressed people’s health situations as pertaining to e-cigarette use (in Vapor Talk Health & Safety) and to quitting without e-cigarettes (in the Stopsmoking subreddit).

### Topic Modeling and Visualization

We trained topic models for four forums: Vapor Talk General E-Cig Discussion, Hookah Forum General Discussion, Vapor Talk Health & Safety, and the Stopsmoking subreddit. We experimented with different numbers of topics in order to find a level of granularity that showed the diversity of discussion topics, while at the same time avoiding topics that were thematically similar. Ultimately, we generated 20 topics for each of the subforums, with the exception of Hookah Forum. Hookah Forum had a greater number of posts than the other forums, as well as a shorter mean post length. With fewer numbers of topics, the themes were not as coherent; thus, we generated 40 topics for Hookah Forum.

We labeled all of these topics and set a minimum threshold for document topics as discussed in the Methods section. In the Stopsmoking subreddit, topics were dispersed in more trace amounts throughout the other posts; thus, it was necessary to lower the threshold to preserve a similar number of document-topics. Aggregate statistics for the four topic models are presented in Table 3.

**Table 3:** Topic modeling results overview.

	Vapor Talk		Reddit	Hookah Forum
	Health	General	Stop-smoking	General
Total posts, n	2376	11,438	2092	17,761
Total topics, n	20	20	20	40
Document-topic threshold, n	0.3	0.3	0.2	0.3
Post length, mean (SD)	487.39 (653.45)	356.35 (447.33)	267.49 (441.77)	323.16 (520.97)

#### E-Cigarette Versus Combustible Cigarette Use

**Figure 2:**
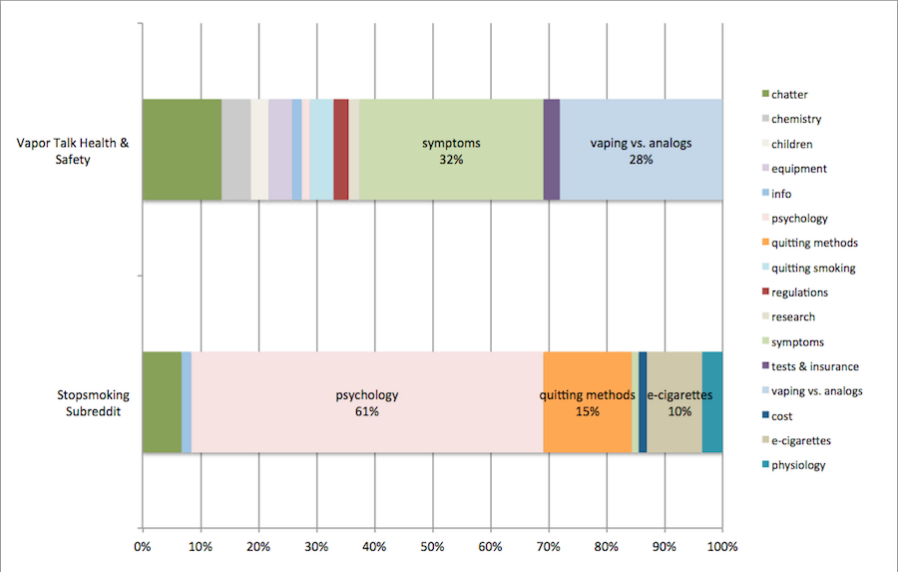
Topic Bars: Quitting in Vapor Talk Health & Safety versus the Stopsmoking Subreddit.

We used the topic modeling results to render a Topic Bars visualization to compare the two forums with the richest discussion of contextual factors: Vapor Talk Health & Safety, and the Stopsmoking subreddit (Figure 2). In Vapor Talk Health, the two most prominent categories were Symptoms and Vaping versus Analogs. With regard to Symptoms, common topics were the health dangers of smoking cigarettes, problems that forum members have encountered in the mouth and throat, the use of propylene glycol (“pg”) as opposed to vegetable glycerin (“vg”), and sleep quality.

In the Stopsmoking subreddit, we saw a much different picture. The most salient bars were Psychology (60.60%, 1435/2368 document-topics) and Quitting Methods (15.29%, 362/2368). In Psychology, the topics discussed included overcoming cravings, dealing with friends, and encouragement that cravings would pass. The Quitting Methods category had only one constituent topic, Quitting Mechanisms, which included terms such as “cold turkey”, “gum”, and “patch”. It is useful to observe that in the Stopsmoking subreddit (Figure 2, bottom), only 9.50% of the discussion content is focused on e-cigarettes (225/2368).

#### E-Cigarettes Versus Hookah

**Figure 3:**
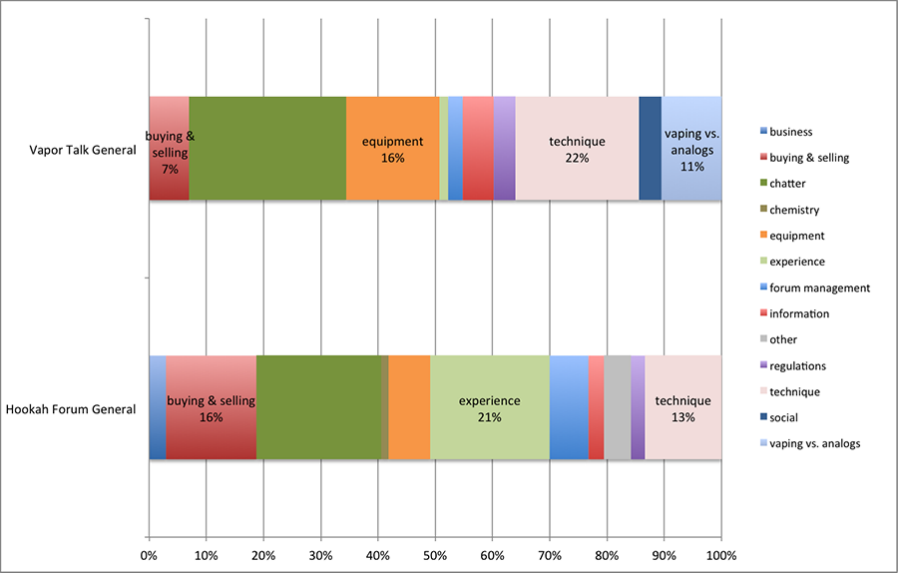
Topic Bars: Vapor Talk General E-Cig versus Hookah Forum General Discussion.

In the last part of our study, we considered the two products: e-cigarettes and hookah. Are these communities different, and if so, how? To consider this question, we compared the Topic Bars visualization for Vapor Talk General E-Cig Discussion and Hookah Forum General Discussion (Figure 3).

There are similarities between the categories of discussions on Vapor Talk and Hookah Forum. In both forums, there was a substantial amount of general chatter (dark green). In addition, both forums featured discussion on buying and selling equipment for e-cigarettes and hookah (red). From the dialogue content, the consumers in Vapor Talk appeared to primarily be end consumers, whereas the consumers in Hookah Forum consisted both of individuals interested in the purchase of hookah equipment for personal use, as well as proprietors of hookah lounges. There were also individuals in both forums whose member type indicated that they were a vendor.

There were also many topics relating to technique (pink). In Vapor Talk, topics concerning technique included how to get a good taste and how different characteristics of the juices affect the vaping experience. In Hookah Forum, sample topics included how to pack the bowl and whether it is a good idea to put other things (eg, alcohol) in the base. Thus, e-cigarette and hookah forums are similar in that their members are actively engaged in information exchange concerning technical and cost-related aspects of the use of their products of choice.

The most prominent difference between Hookah Forum and Vapor Talk is the greater focus on equipment in Vapor Talk (orange), as opposed to the focus on the use experience in Hookah Forum (light green). In Hookah Forum, there is a great deal of discussion of different flavors, “buzz”, and clouds. A large proportion of Vapor Talk is devoted to equipment, that is, discussion of the different types and parts of e-cigarettes, including mods, tanks, coils, atomizers, cartomizers, and batteries.

There is some discussion in these two forums about health—a substantive part of the conversation in Vapor Talk focuses on vaping as opposed to smoking “analogs” (traditional cigarettes), and though not as prominent in the discussion content, a number of health concerns were also expressed in Hookah Forum, relating to headaches, lung issues, and vocal chord problems. There was also discussion on ways to prevent getting sick from smoking hookah, including eating prior to smoking and staying properly hydrated—though one might consider this not a matter of health concern, but rather, a practical consideration in order to enjoy the experience.

## Discussion

### Principal Findings

In this paper, we used text mining and visualization techniques to examine the use of different tobacco products. At the outset, we identified contextual factors of these behaviors, particularly in terms of health impacts and concerns. Then we generated a heat map that enabled us to compare forum content in terms of these factors of interest. Based on this information, we selected two forums that contained the highest densities of these factors and rendered a topic modeling-based visualization, Topic Bars, to compare these forums. This comparison enabled us to gain insights concerning the experience of tobacco use with e-cigarettes and the experience of tobacco use without e-cigarettes. Last, we constructed another Topic Bars visualization to compare general e-cigarette and hookah discussion, to investigate similarities and differences between the communities.

The main contributions of this paper are as follows. First, we have demonstrated an approach using text mining and visualization techniques to select particular social media datasets out of a larger pool, for a particular health behavior. The crux of this technique is to identify factors of interest for developing strategies to facilitate behavioral change and then employ relevant lexicons to assess and compare the amount of content concerning these factors, across datasets. This technique can be helpful for characterizing discussion forums as a whole, as well as in the selection and differentiation of social media datasets to investigate specific research questions.

Second, this paper shows that e-cigarettes provide a very different experience of tobacco use as compared to combustible cigarettes. When smokers who are trying to quit visit a discussion forum, they report on the difficulties they are having trying to quit, and others in the forum chime in to offer their encouragement. The psychological element is extremely salient, and the focus is on quitting. In the case of e-cigarettes, we saw that much of the discussion focused on symptoms that people were experiencing as they were using e-cigarettes. People using e-cigarettes appear less likely to engage in the psychological battle of quitting. The e-cigarette has diverted their attention to a different activity, dealing with concrete problems to avert particular physiological symptoms associated with e-cigarette use. Moreover, at least for some Vapor Talk users, their goal is to be analog free rather than nicotine free, and hence a psychological struggle is less evident.

The difference in psychological state and engagement of the consumer is an important concern on two levels. In terms of regulation and policy concerning electronic cigarettes, there are no clear answers, but the findings of this study highlight the importance of considering impacts on psychological state and engagement in the regulation of electronic cigarettes as opposed to combustible cigarettes. On an individual level, users of tobacco products interact with electronic cigarettes in very different ways than they do combustible cigarettes, and thus, the pathway that one faces in quitting the use of all tobacco products appears to be fundamentally different. Counselors and those designing educational programs designed for smokers should be aware of the differences so that they can provide different types of support to facilitate changes in health behavior.

Last, this study examined the general content in discussion forums for e-cigarette and hookah. There are strong similarities, and ultimately, both are focused on improving the use experience, which has a strong sensory component. These are “hobbyist cultures” in that their members are enthusiastic users and sharers of information concerning their common activity. Particularly given the rapid rate at which the two products are growing in popularity, online communities, as common sites of information diffusion and as sources of the latest information, are ideal environments to study both.

### Limitations

This work has various limitations. First, we harvested data from three websites, and there are certainly many other online communities relating to tobacco products. We deliberately selected different types of communities and subsetted the communities in order to examine similarities and differences within and between communities. As we expected, the selected communities vary in many characteristics, suggesting that they represent a range of tobacco users’ experiences. However, this investigation focused on a subset of online communities that are available to users of tobacco products, and it would be valuable to examine additional communities in the future, for example, by comparing multiple forums for e-cigarettes and/or multiple forums for hookah in order to characterize the variability in topics addressed in online communities for the same product type. Additional research might also consider the content in relation to the demographic characteristics of the users, which was out of the scope of the current study.

Second, the users in an online community are not necessarily representative of users of tobacco products, cigarettes, e-cigarettes, and/or hookah in general. While we agree that this is true, today, if a typical user goes to a search engine and types “e-cigarette sore throat”, among the first entries to come up would be links to specific threads on this subject in discussion forums including Vapor Talk. Thus, the potential for exposure to a much larger number of people, those who do not actively participate in discussion forums, is a reality.

Third, in this study we constructed lexicons to assess contextual factors of interest for a particular type of behavior with health impacts. The lexicon is not necessarily generalizable to other types of health behaviors, nor would it necessarily perform comparably over time. It is likely that as language evolves, the lexicon would need to be augmented. However, there is potential here to extend the lexicon for application to other health contexts and time periods.

###  Atmosphere of the Forums and Implications

In this paper, we employed two primary techniques, a contextual heatmap, and a Topic Bars visualization, in order to explore differences between data sets. The Topic Bars visualization enabled us to specifically compare different discussion forums. We now consider some of the differences in topics between forums and what this may mean.

The results of the topic models on Vapor Talk Health and Stopsmoking subreddits suggest that those who attempt to quit smoking combustible cigarettes and those who use e-cigarettes have very different experiences. It appears that many who use e-cigarettes encounter problems that may lead them to do research and perhaps find a solution; thus, the forums contain detailed accounts of the technical intricacies of vaping and the health issues that may be encountered. Though a minority of the members of the Stopsmoking subreddit appear to use e-cigarettes, for the most part this group appears to take more traditional approaches to quitting, with emphasis on mutual encouragement and support, and coping with the psychological aspects of this experience. These topic modeling results suggest that, without e-cigarettes, the aspect of quitting that is most salient is the psychological hurdle, though it is important to state that users may be using e-cigarettes but not reporting this activity in their Stopsmoking subreddit discussion.

The information exchanged and atmosphere of support in these two forums appears to be quite different. Whereas Vapor Talk includes detailed reports of symptoms and their temporal context (eg, how long the symptoms have lasted and when they started), the Stopsmoking subreddit appears to be focused on mutual encouragement, reinforcement of the value of quitting, and strategies for overcoming cravings. Time is important here also, but the nature of that time is different. Many forum participants report how long it has been since they quit, and others add words of encouragement and how long it has been since they quit. Thus, there are many shorter posts here.

The interactions in the two forums have both similarities and differences to existing literature on online support groups for smoking cessation. Previous studies of discussion forums for quitting smoking have found that most participants were women, and that they used the forum mostly as a source of emotional support and encouragement, and less often for the purposes of eliciting practical information and quitting tips [46,47]. Consistent with this work, there appeared to be a substantial amount of support and encouragement. However, in contrast to prior work, there did appear to be information and quitting tips exchanged. In Vapor Talk Health & Safety, the tips often took the form of concrete advice about the types of e-liquids to use, how to inhale, and so on, which could potentially alleviate problems with the mouth and throat. In the Stopsmoking subreddit, the tips were often psychological, concerning how to overcome the desire to smoke.

In our topic modeling results comparing e-cigarette and hookah discussion (Vapor Talk and Hookah Forum), initially there appear to be substantive differences in the content. However, there are similarities in the nature of the communities. In the case of hookah, the use experience is prominent, including discussion of the “buzz”, smoke rings, and clouds. In the case of e-cigarettes, the equipment and techniques features more prominently, but much of that discussion is on how to get a “throat hit” or a better taste. Thus, improving the experience is a common theme in both forums.

In summary, the results of these two topic models suggest similarities in the e-cigarette and hookah general discussion. Both are communities composed of enthusiastic users of a product who are actively engaged in the discovery and sharing of new information on how to obtain or enjoy the products that they champion. As such, this content can be invaluable in terms of providing knowledge of the day-to-day use problems that may occur with the two products.

## Appendix

Multimedia Appendix 1 Factors of tobacco use lexicon.

Multimedia Appendix 2 Additional stop words.

Multimedia Appendix 3 Topic modeling results.

## Abbreviations

LDA: Latent Dirichlet Allocation
