# Supplementary material for: What Online Communities Can Tell Us About Electronic Cigarettes and Hookah Use: A Study Using Text Mining and Visualization Techniques
Source: J Med Internet Res. 2015 Sep 29;17(9):e220. doi: 10.2196/jmir.4517 (PMC4642380; doi:10.2196/jmir.4517)
Supplement: Multimedia Appendix 2 [file jmir_v17i9e220_app2.pdf]

review
quote
badge
date
message
stopsmoking
body
badges
extra
reddit
reset
community
users
calendar
dd
mm
yyyy
subject
format
badgebot
messages
iphone
january
february
march
april
may
june
july
august
september
october
november
december
jan
feb
mar
apr
may
jun
jul
aug
sep
oct
nov
dec
edit
edited
pm
don
hadn

didn
didnt
doesnt
wouldn
wasn
weren
aren
shouldn
couldn
id
isn
ill
ll
ive
ve
haven
doesn
dont
im
lol
lot
